# Growth Dynamics of Ultrathin Films of Benzo[1,2-*b*:4,5-*b*’]dithiophene Derivatives on Au(111): A Photoelectron Spectroscopy Investigation

**DOI:** 10.1021/acs.langmuir.3c00572

**Published:** 2023-04-07

**Authors:** Angelo Stummo, Monica Montecchi, Francesca Parenti, Davide Vanossi, Claudio Fontanesi, Raffaella Capelli, Luca Pasquali

**Affiliations:** † Department of Engineering “Enzo Ferrari”, University of Modena and Reggio Emilia, Via Pietro Vivarelli 10, 41125 Modena, Italy; ‡ Department of Chemical and Geological Sciences, University of Modena and Reggio Emilia, Via Campi 103, 41125 Modena, Italy; § IOM-CNR, Strada Statale 14, Km. 163.5 in AREA Science Park, Basovizza 34149, Trieste, Italy; ∥ Department of Physics, University of Johannesburg, P.O. Box 524, Auckland Park 2006, South Africa

## Abstract

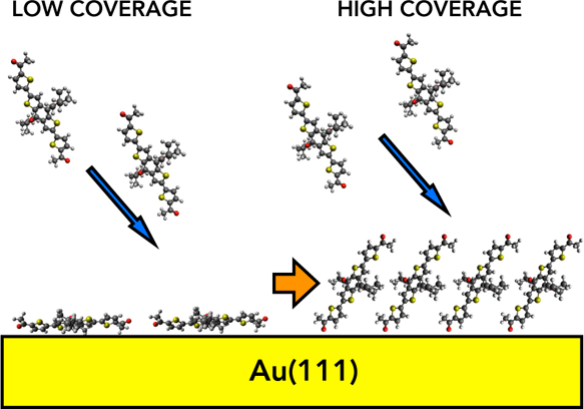

Ultrathin films of a stereoisomeric mixture of benzo[1,2-*b*:4,5-*b*’]dithiophene derivatives were grown by thermal evaporation in vacuum on Au(111), and they were studied in situ by photoelectron spectroscopy. X-ray photons from a non-monochromatic Mg Kα conventional X-ray source and UV photons from a He I discharge lamp equipped with a linear polarizer were used. He I photoemission results were compared with density functional theory (DFT) calculations: density of states (DOS) and 3D molecular orbital density distribution. Au 4f, C 1s, O 1s, and S 2p core-level components suggest a surface rearrangement as a function of film nominal thickness, with the variation of the molecular orientation, from flat-laying at the initial deposition to tilted toward the surface normal at coverages exceeding 2 nm. Eventually, the DFT results were exploited in assigning of the valence band experimental structures. Moreover, polarization-dependent photoemission confirmed the tilted arrangement of the molecules, starting at 2 nm. A variation of the work function of 1.4 eV with respect to the clean substrate was measured, together with a valence band offset of 1.3 eV between the organic layer and gold.

## Introduction

Thiophene-based molecules, and in particular benzodithiophene (BDT) derivatives, are recognized as important organic semiconductors for (opto)electronic devices and for photovoltaic energy conversion, thanks to their easy chemical synthesis, good fabricability in solid state, good electrical transport efficiency, electro-optical properties (light absorption and emission and excitonic effects), and good stability when exposed to environmental conditions.^1−7^ Electronic structure such as the energy value of the highest occupied molecular orbital (HOMO) and lowest unoccupied molecular orbital (LUMO), as well as electron or hole doping, optical properties, molecular packing in the solid state, and molecular ordering can be tailored, thanks to functionalization with appropriate chemical groups. The conjugated main core of BDT determines the position of the Frontier molecular orbitals. The addition of alkoxy chains or conjugated groups bound to the two benzenic para positions and/or the modification of the terminal groups along the BDT longitudinal axis, apart modifying the electronic structure, can contribute sizably to molecular solubilization as well as molecular packing and film morphology in the solid state.^1,8−11^ Besides this, these molecules appear to be quite stable and appropriate for thermal evaporation under controlled conditions. This allows one to produce ultrathin films of high purity and distinctive ordering, which can be fruitfully exploited in the fabrication of thin-film electronic devices.^6,12^

**Figure 1:**
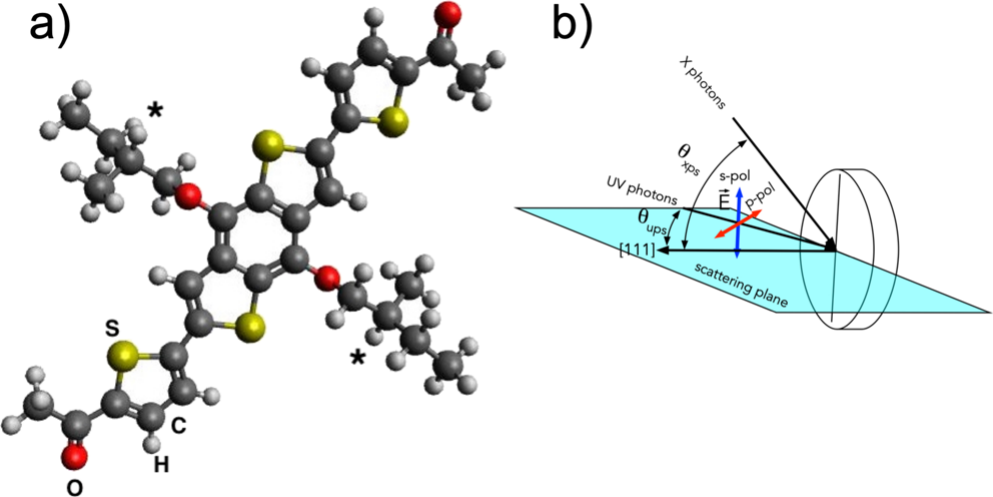
(a) Ball and stick BDT-COR molecular structure (a stereoisomeric mixture was used): carbon, oxygen, sulfur, and hydrogen atoms have been represented by gray, red, yellow, and white colors, respectively; chiral centers have been labeled with an asterisk; (b) scheme of the experimental geometry set-up with reference to the incoming photons.

In the present work, we have grown ultrathin films of a benzo[1,2-*b*:4,5-*b*’]dithiophene derivative (BDT-COR)^13^ on Au(111), by molecular beam epitaxy. BDT-COR is a BDT derivative of original synthesis where the BDT core is functionalized in the benzene para positions with 2-methylbutyloxy side chains and in the thiophene alpha positions with thienyl spacers bearing electron acceptor acetyl groups. The BDT-COR molecule presents a typical acceptor-π-donor-π-acceptor architecture and is schematized in Figure 1a.

Moreover, since the alkoxy chains have been inserted by the reaction of commercially available benzo[1,2-*b*:4,5-*b*’]dithiophene-4,8-dione with a racemic bromo alkane precursor, the BDT-COR studied here is a stereoisomeric mixture containing the two diastereoisomeric forms (*R*,*R* and *S*,*S*) and the meso compound (*R*,*S*) (see Figure 1a, where asterisks denote the chiral centers).

In the case of the enantiopure form, the presence of chiral centers in the alkoxy side “wings” can play an important role in the molecular arrangement in the solid state and/or in the possibility of emission of light with circular polarization.^14−20^

The details of molecular synthesis, optical properties, as well as the possibility to form thin films by thermal evaporation on SiO_2_ and polymethyl methacrylate (PMMA) substrates have been presented elsewhere.^13^ It has been observed that BDT-COR exhibits interesting properties such as broad absorption and emission bands in the visible region and a good thermal stability that makes it possible to grow thin films by thermal deposition. In the previous study by some of us, an enantiopure sample of BDT-COR exhibited a clear circular dichroism.^13^ In spite of that, the so-evidenced chiral character was not accompanied by the formation of homogeneous and continuous thin films, regardless of the surface chemical composition of the substrate.^13^ This deeply undermines the possibility to exploit the enantiopure BDT-COR phase in electronic devices, for example, for applications based on the chiral-induced spin selectivity effect.^21^ On the contrary, BDT-COR as a stereoisomeric mixture demonstrated a layer-by-layer growth mechanism when evaporated on PMMA.^13^ Moreover, it shows optimal orientation for enabling the charge transport in the field-effect geometry typical of transistors, being the molecular long axis perpendicular to the substrate.^13,22^ On PMMA, atomic force microscopy (AFM) evidenced the formation of highly homogeneous and continuous molecular films at the nanoscale level, as required for efficient charge transport. When the growth of the BDT-COR mixture was carried out on SiO_2_ instead, an ensemble of isolated and distant grains was obtained, which is completely inappropriate for electronics applications. In order to properly evaluate the potential of the BDT-COR stereoisomeric mixture in organic electronics and considering the critical dependence of the thin-film molecular aggregation of this compound on different substrates, the study of the interaction with a metal is extremely relevant. Indeed, for any electronic device, the metal surface acts as a charge injection interface, and for a large number of specific architectures, the metal is also used as the supporting substrate of the molecular active layer. For all these reasons and considering a representative reference metal as, for example, Au or Ag, the molecular arrangement as well as the alignment of the HOMO and LUMO levels of BDT-COR with respect to the metal Fermi level should be taken into account, to have a clearer picture of the potential behavior of this class of molecules in electronics.

Here, we study the interface formation of BDT-COR (stereoisomeric mixture) on Au(111), that is the mostly used and prototypical metallic substrate for molecular electronics. We performed in situ photoelectron spectroscopy analyses, namely, X-ray photoelectron spectroscopy (XPS) and ultraviolet photoemission spectroscopy (UPS), also exploiting the orientation of the electric field direction of linearly polarized incident light with respect to the sample axes, to obtain information on the molecular reactivity, chemical composition, interface electronic states, energy level alignment, and molecular orientation. Electron spectroscopy experiments were accompanied by density functional theory (DFT) simulations of the electronic structure of the molecule, to guide the interpretation of the experimental results.

## Experimental Section

The Au(111) single crystal (Mateck) was cleaned by repeated cycles of Ar^+^ sputtering (0.5 keV at grazing incidence) and annealing (up to 450 °C) until valence band photoemission revealed the appearance of typical surface states and surface resonances of the clean gold.^23−26^ Surface cleanliness was also checked by inspection of the Au 4f and C 1s core levels by XPS. The base pressure was 2 × 10^–10^ mbar. BDT-COR molecules, synthesized as reported elsewhere,^13^ were thermally evaporated in ultra-high-vacuum, with a cell equipped with a quartz crucible and at a temperature of 175 °C. The film thickness was monitored with a quartz microbalance. The Au substrate was kept at room temperature during growth. In the following, film thickness is expressed in terms of nominal thickness, corresponding to the effective thickness as measured by the quartz microbalance. The growth rate was 0.2 nm/min. The effective thickness was also checked in a separate experiment by AFM on a BDT-COR film grown on PMMA.^13^

The photoemission signal was measured using a hemispherical electron analyzer (Omicron EA125) working at constant pass energy and at normal emission. For XPS acquisition, the Mg Kα radiation at 1253.6 eV from a dual-anode non-monochromatic X-ray source (VG Microtech-XR3) was used, operated at 15 mA and 15 kV. Experimental geometries are shown in Figure 1b. The angle between the X-ray source and the detection direction (along the [111] surface normal of gold) was θ_xps_ = 49.2°. Survey scans were acquired with a resolution of 1 eV. Spectra corresponding to the main photoemission peaks of the constituting elements were taken with a resolution of 0.5 eV. The energy scale of the spectra was calibrated considering the position of the Au 4f_7/2_ peak of the clean substrate at 83.9 eV of binding energy. For UPS, a high-intensity, dual-stage differentially pumped discharge source (VG Microtech-UVL-HI) was used. UPS was performed with He I photons (21.2 eV) and a resolution of 50 meV. The UV impinged at θ_ups_ = 45° with respect to the surface normal. The UV source was equipped with a three-mirror linear polarizer, which allows one to select either unpolarized or linearly polarized light, under s- or p-scattering conditions (i.e., with the electric field of the impinging light either perpendicular or parallel to the scattering plane containing the surface normal, respectively, as represented in Figure 1b).

Work function values were evaluated by considering the relative position of the low-kinetic-energy cutoff of the UPS spectra, as obtained by linear extrapolation, with respect to the emission from the Fermi-level position. This was done after application of a negative bias of a few volts to the sample to shift the photoemission spectra rigidly on the kinetic energy scale. The work function was then obtained as ϕ = *h*ν – (*E*_K,Fermi_ – *E*_K,cutoff_), where *E*_K,Fermi_ was the kinetic energy of the electrons emitted at the Fermi level and *E*_K,cutoff_ was the kinetic energy of the cutoff of the tail of the secondary electrons.

## Theoretical Methods

The equilibrium geometry, electronic structure, and density of states (DOS) of a single BDT-COR molecule were calculated by DFT with the computer code STOBE.^27^ A gradient-corrected RPBE exchange/correlation functional was used.^28,29^ In the case of sulfur, oxygen and carbon centers an all-electron double-valence plus polarization DZVP Gaussian basis was used, while a (311/1)-type basis was chosen for the hydrogen centers.^27^

## Results and Discussion

**Figure 2:**
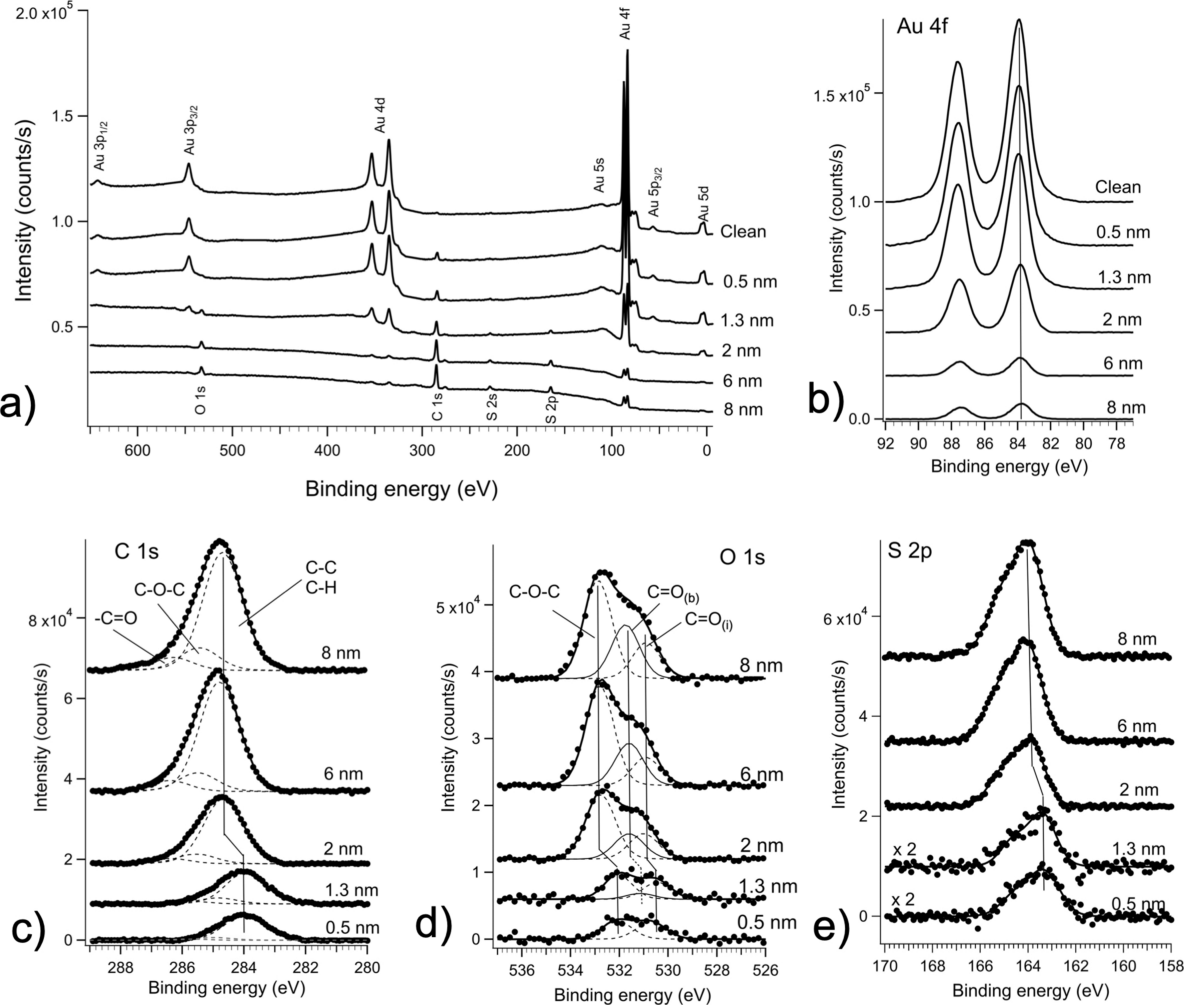
(a) Survey XPS scans recorded with Mg Kα photons as a function of nominal coverage. Details of the Au 4f (b), C 1s (c), O 1s (d), and S 2p (e) spectra are also shown, together with their decomposition into multiple components, through a best-fit analysis with Voigt profiles.

The survey XPS spectra acquired on films of increasing nominal thickness are reported in Figure 2a.

Analyses of the survey scans as a function of coverage reveal the progressive appearance of the characteristic elemental peaks of the BDT-COR molecule, which increase in intensity together with the increase of the film thickness. Correspondingly, the characteristic features of the Au substrate gradually decrease. This is more clearly visible in the spectra of the different core levels of interest taken at a higher resolution, as shown in panels (b–e) of Figure 2 after a preliminary subtraction of the Mg source satellites and a Shirley-type background. While the position of the Au 4f peaks remains fixed at all coverages (Au 4f_7/2_ at 83.9 eV), it can be noticed that the peaks characteristic of BDT-COR, that is C 1s, O 1s, and S 2p tend to shift toward higher binding energy, after the initial deposition stages and for coverages thicker than 1.3 nm. In particular, the shift of the C 1s maximum is +0.7 eV, when passing from a nominal coverage of 1.3 to 2 nm, and then it remains constant. An analogous result is also observed for the O 1s and S 2p structures. The high-resolution spectra have been decomposed into the main constituting components through a best-fit analysis with Voigt profiles. Au 4f (Figure 2b) shows a constant single doublet, corresponding to the Au 4f_7/2_ and 4f_5/2_ spin–orbit split components, separated by 3.67 eV, typical of gold. The C 1s spectrum presents a slightly asymmetric lineshape that has been fitted with three Voigt components. The most intense component at 284 eV (which shifts to 284.7 eV for thicknesses larger than 1.3 nm) is associated with carbon atoms bonded to either carbon atoms or hydrogen atoms in the BDT-COR molecule. The higher binding energy structures are associated with carbon atoms singly or doubly bonded to oxygen, respectively.^30^ This reflects the molecular structure, in which there are two types of bound oxygen atoms: two oxygen atoms forming single bonds with two carbon atoms at both sides, in the two alkoxy side chains, and two oxygen atoms double bonded to one carbon atom (carbonyl group) each in the end groups (Figure 1a). The relative branching ratio between the two associated C 1s components, approximately 2:1, correctly reflects the stoichiometry of the involved chemical groups. Concerning O 1s, apart from the already mentioned progressive shift with coverage, the best-fit decomposition indicates a more complex scenario. The spectrum acquired at 0.5 nm of coverage has been fitted with two components of the same weight. The low-binding-energy component is associated with oxygen atoms in C=O terminals and the high-binding-energy component is associated with the C–O–C oxygen atoms of the side alkoxy chains.^30^ The subscript (i) has been used to indicate atoms located at the interface with the gold substrate. At higher coverage, these components shift toward higher binding energy. In order to fit the spectra with the appropriate separation between the two C=O and C–O–C components, of about 1 eV as reported in literature for these chemical units,^30^ a new component was introduced. This component has been labeled C=O_(b)_ and is associated with “bulk” like C=O oxygen atoms, that is, to oxygen atoms located far from the interface plane.

The S 2p spectra shown in Figure 2e have been fitted with one Voigt doublet, accounting for the spin–orbit splitting of 1.2 eV between the 2p_3/2_ and the 2p_1/2_ components. As observed, the 2p_3/2_ maximum shifts from 163.4 eV at early coverage, to 163.9 eV at 2 nm and above.

**Figure 3:**
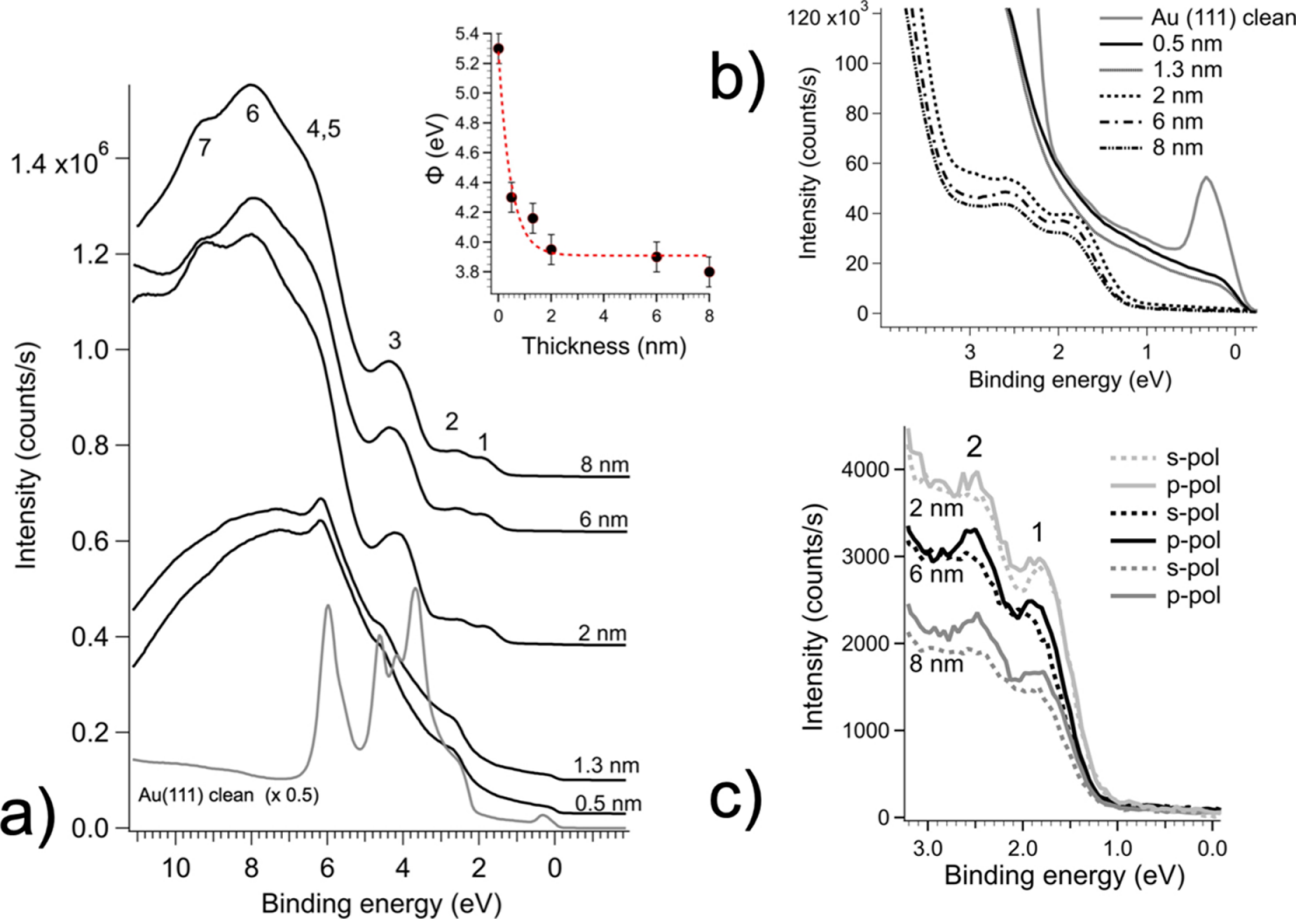
(a) UPS spectra taken with He I unpolarized photons, *h*ν = 21.2 eV, as a function of the nominal coverage of BDT-COR; the inset shows the variation of the work function with coverage, as calculated from the low-kinetic-energy cutoff of the electron distribution curves (not shown); the dashed red line serves to guide the work function evolution as a function of the thickness; (b) a closer inspection of the top of the valence band region; and (c) the top of the valence band taken with linearly polarized photons in s- and p-scattering geometries for the 2, 6, and 8 nm thick films.

UPS spectra taken as a function of nominal film thickness are shown in Figure 3a. The spectrum of clean Au(111) presents the typical—slightly asymmetric—surface state at about 0.35 eV of binding energy, referred to the Fermi energy.^24−26^ This is more clearly evidenced in the magnification of the valence band top shown in Figure 3b. The 5d band develops between 2 and 7 eV, showing the characteristic fine structure typical of the clean Au(111) surface, superimposed with the specific surface resonances.^23,25,26^

At early deposition stages, the surface state features of the clean gold are completely smeared out, and also the 5d band is strongly depressed. The position of the Fermi level is still clearly distinguishable. Distinctive and resolved structures associated only with the BDT-COR overlayer are difficult to spot. A broad emission shoulder is observed below the Au 5d band, between 2 eV and the Fermi energy, and a broad undefined structure also shows up above the 5d band, above 6.5 eV.

The results are markedly different for the 2 nm thick film and for the subsequent coverages. At this stage, the Au substrate features cannot be detected, and well-defined structures of the molecular layer are observed. These have been labeled with progressive numbers in Figure 3.

The inset of Figure 3 shows the evolution of the work function as a function of coverage. The work function of the clean surface, which is 5.3 ± 0.1 eV, reaches a saturation value of 3.9 ± 0.1 eV already at 2 nm film thickness. It should be noted that above this stage also, the overall features of the valence band have reached a line-shape plateau, which remains constant as the BDT-COR surface coverage is increased.

Valence band spectra have also been acquired with linearly polarized He I photons with the electric field either perpendicular to the scattering plane (s-polarization, see Figure 1b), that is with the electric field vector parallel to the sample surface, or parallel to the scattering plane (p-polarization, see Figure 1b), that is with a component of the electric field vector perpendicular to the sample plane. The most pronounced differences between the two polarizations are observed at the valence band top (Figure 3c) and in particular for the molecular states labeled 1 and 2. It can be observed that these features are better defined with the light in the p-type configuration. At higher binding energy and at coverages less than 2 nm, it was not possible to detect significant differences between the two polarizations and the data are not reported.

From the XPS and UPS results, it appears that a variation in the film properties takes place after the earliest stages of deposition, namely, above a coverage of 1.3 nm. In this work, we did not proceed intentionally to evaluate the effective thickness of the deposited layers through the analysis of the attenuation of the core levels of the substrate,^31−34^ since the final morphology, homogeneity, and thickness uniformity of the films are not known and the evaluation of the specific inelastic mean free paths could lead to sizable errors. For this reason, we preferred to express the thickness in terms of values derived just from the quartz microbalance measurements. A standard evaluation of the effective film thickness on the basis of the attenuation of the gold core levels has been attempted, yielding results that are compatible with those of the quartz microbalance up to about 2 nm. Sizable deviations at higher coverage were observed, which could be related to the development of three-dimensional islands.

In XPS, it is observed that all core levels appear at a reduced binding energy at the early deposition stages, with respect to the bulk molecule. This can be related to an enhanced screening of the core hole in the photoemission process due to the proximity to the metal substrate at the interface.^33,35^ The fact that all core levels are involved in the shift seems to indicate that the molecules tend to adopt a planar arrangement. In particular, the separation of 1.4 eV between the oxygen components related to C=O and C–O–C at coverages of 0.5 and 1.3 nm (Figure 2d) is slightly higher with respect to the values reported in the literature.^30^ This could be related to the end-terminal oxygen atoms being closer to the substrate, thus experiencing a more pronounced screening with respect to the oxygen atoms in the alkoxy side chains. This, in turn, suggests a slight molecular distortion with respect to the planar configuration, mainly associated with the alkoxy chains. At 2 nm of coverage and above, a new component of O 1s related to the C=O terminal group appears, in this case featuring the correct energy distance to the C–O–C component. Correspondingly, a rigid shift of all spectral features occurs toward higher binding energy. This new scenario is ascribed to a change in the molecular orientation of BDT-COR in the adsorbed film. The new intermediate O 1s component, at about 531.5 eV, is associated with C=O far from the substrate, that is not screened by the metal substrate. This has been labeled as C=O_(b)_ in Figure 2d to suggest it as being “bulk”-like in nature. It should be noted that this component can be already detected in the 1.3 nm spectrum. The lower-binding-energy component, labeled C=O_(i)_, is still associated with atoms close to the interface but those that are exposed to a reduced screening from the substrate with respect to the case of BDT-COR lying flat on the substrate. This could be related to a slightly higher distance from the substrate and/or to a change in the orientation of BDT-COR, upon increasing the surface molecular density, that is, on increasing the surface coverage. This is consistent with the position of the C–O–C peak, which, above a coverage of 2 nm, is not subject to screening effects by the substrate. Therefore, at a coverage of 2 nm, a variation in the orientation of the molecules becomes apparent, with their long principal axis tilted away from the substrate plane toward the sample normal, with one C=O terminal closer to the substrate and the other pointing outward. At 2 nm, the two C=O_(i)_ and C=O_(b)_ O 1s components present a similar spectral weight, which could correspond roughly to a layer of tilted molecules. At higher coverage, the spectral weight of C=O_(b)_ progressively increases, that is compatible with both a more packed (and more tilted) molecular configuration, damping the interface feature with respect to the bulk one due to the different peak-intensity attenuation for emitting centers at different depths (for O 1s photoemission peaks in organic layers excited with Mg Kα photons, the inelastic electron mean free path corresponds to about 2 nm^36,37^), and the formation of multiple layers. At this stage, this can be associated also with the development of three-dimensional islands of tilted molecules, on top of a continuous layer of (tilted) molecules wetting the substrate. The persistence of the C=O_(i)_ component up to 8 nm supports this idea. The intensity of the C–O–C component does not suffer intensity variations, confirming the equivalent contributions of the two oxygen atoms in the alkoxy chains. Regarding the sulfur 2p spectra shown in Figure 2e, they have always been fitted with a single 2p doublet, accounting for only one sulfur configuration in the molecule. Its energy position is consistent with sulfur in thiophene rings, with no indication of molecular fragmentation or strong bonding with the substrate.^38−41^ As long as the film thickness increases, the energy shift toward higher binding energy is consistent with the reduced screening from the substrate. Moreover, the fact that the S 2p low-binding-energy interface component is not present above 2 nm is consistent with molecules that change their orientation and are no more parallel to the substrate plane. This behavior is also in agreement with the analogous behavior of the C 1s levels shown in Figure 2c.

**Figure 4:**
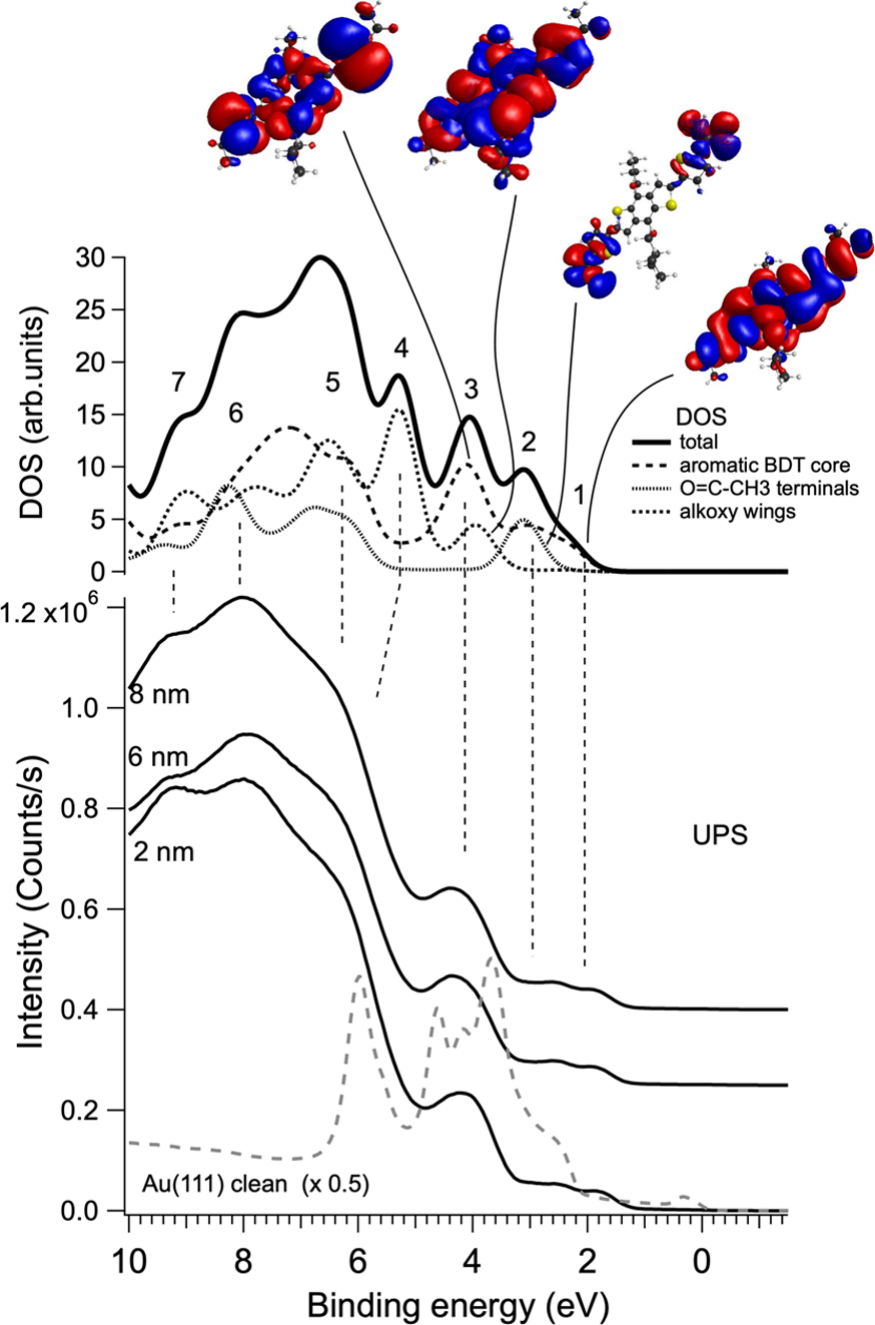
Comparison between total and partial DOS and the experimental valence band. The contour plot of significant molecular orbitals has been also reported.

Regarding the valence band, it is observed that this is almost completely developed and structured, with no more contributions from the substrate, starting from 2 nm of nominal coverage. Remarkably, 2 nm also corresponds to the length of the molecule along its longer axis. At this stage also, the Fermi level is not visible in the spectra. This scenario is, therefore, compatible with a relatively uniform film of “standing” BDT-COR molecules, presumably tilted with respect to the substrate normal, in agreement with the XPS results, which suppress the emission from the substrate in UPS. Above 2 nm, the valence band evolves only slightly. This is further confirmed by the variation of the work function, which reaches saturation at a coverage of 2 nm. To support the interpretation of the spectral features, we calculated by DFT the DOS of BDT-COR.^33,34,42^ The comparison between the experimental valence band and the total and partial DOS is shown in Figure 4. For comparison purposes, the DOS has been shifted rigidly on the energy scale to align with the UPS curves. An overall correspondence can be found between the experimental features and the calculated DOS. This indicates that the solid state does not substantially modify the molecular electronic orbitals of the single molecule. The structure labeled 1 is associated with the BDT-COR HOMO and has a π character mostly localized over the central conjugated core. Feature 2 is due to the superposition of π-type states, again localized mainly on the aromatic framework, and σ-type states due to the carbonyl moieties. Alkoxy wings start contributing to feature 3, where their spectral weight superimposes with other π-type states of the BDT-COR core. At higher binding energies, that is, for structures 4–7, contributions from all portions of the molecule tend to sum up. Besides the assignment of the spectral structures, the contour plots of the molecular orbitals contributing to the low-binding-energy features 1 and 2 are also extremely useful for the interpretation of the spectra acquired with different light polarizations, as shown in Figure 3c. In fact, the direction of the light polarization vector in photoemission can be exploited to obtain information on orbital symmetry, due to the directionality of the dipole matrix element during photoexcitation.^43^ Both structures appear well resolved in the spectra acquired under p-scattering conditions. This effect is even more pronounced for feature 2, which exhibits contributions of σ character due to the C=O terminals. These findings strongly support the idea that above a coverage of 2 nm, the molecules are not parallel to the substrate and adopt a tilted configuration, with one of the C=O–CH_3_ end groups pointing outward, in agreement with the XPS results.

Changes in the molecular orientation with respect to the film thickness have already been observed in prototypical π-conjugated organic systems, such as pentacene^22^ and α-sexithiophene (T6) molecules^44^ grown on SiO_2_. For very low film coverage, that is, before the formation of a complete layer, these systems demonstrated a competition between the intermolecular π–π interaction, which promotes a molecular packing that results in the long axis of each molecule being almost perpendicular to the substrate and maximize the π–π core overlap, and the molecule–substrate interaction, which promotes a flat-lying orientation. For pentacene, a progressive increase of the molecular tilt toward the surface normal has been reported as a function of increasing film thickness.^22^ Differently, for T6 at low coverages, a distribution of regions of molecules either perpendicular to the substrate or completely flat have been observed.^44^ By increasing the film thickness/number of molecules in the films, the π–π interaction started prevailing and, at high coverages and for both pentacene and T6, the molecules changed their initial orientation, and became completely perpendicular to the substrate.

**Figure 5:**
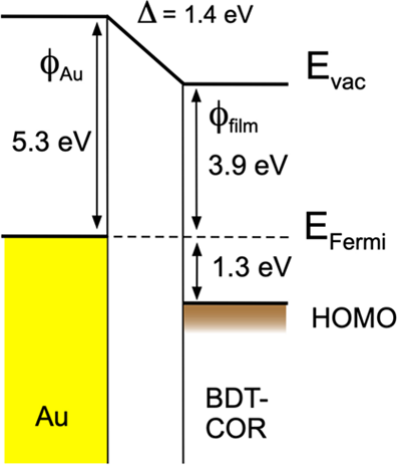
Energy diagram of the BDT-COR/Au interface.

Eventually, from the linear extrapolation of the low-binding-energy cutoff at high coverage, the valence band offset with respect to the Fermi level can be obtained. Valence band offset between gold and BDT-COR is 1.3 ± 0.1 eV. The energy diagram of the interface, including variation of the work function, is presented in Figure 5, where the work function of the gold surface and that of the thick film are also indicated, as derived from Figure 3.

## Conclusions

X-ray and UV photoelectron spectroscopies were used to study the electronic structure and the molecular arrangement of the BDT-COR/Au(111) interface, as a function of the molecular layer thickness. BDT-COR is a promising BDT derivative of the original synthesis bearing thienyl spacers with acetyl groups and 2-methylbutyloxy side chains anchored in the para position to the central benzene unit. The molecules form stable films at room temperature, without fragmentation. Core levels of BDT-COR and valence-band spectral dependence upon surface coverage indicate a variation of the molecular organization occurring at a nominal thickness of about 2 nm. While at the earliest deposition stages, the analysis of the core levels of the molecule indicates that BDT-COR tends to adsorb flat on the Au(111) surface, without forming strong bonds with the substrate, at increasing coverage, a change in the orientation is observed, with the molecules tending to tilt toward the substrate normal. This variation of the molecular orientation is presumably due to an increased molecular density favored by the π–π intermolecular interactions (stacking). This is also accompanied by a change in orientation, at coverages exceeding 2 nm, by the possible development of three-dimensional islands on top of a tilted molecular film. The interpretation of the valence band features is supported by DFT calculations of the DOS of the molecule and the associated molecular orbitals. The DFT results confirm the spectral dependencies of the topmost molecular orbitals on the direction of the light polarization vector in UPS with respect to the substrate plane. Polarization-dependent UPS also suggests that the molecules above a coverage of 2 nm tend to tilt toward the substrate normal, in a “standing” orientation with respect to the Au(111) surface. The work function variation was also monitored, indicating that a saturation value is reached at about a coverage of 2 nm, corresponding to the change in the molecular orientation. The valence band offset between Au and BDT-COR is evaluated to be 1.3 eV.

## Abbreviations

XPS: X-ray photoelectron spectroscopy
UPS: ultraviolet photoelectron spectroscopy
DFT: density functional theory
DOS: density of states
